# Temporal dynamics of microglial neuroprotection during chronic Aß ‐treatment in human iPSC‐derived 3D neurospheres

**DOI:** 10.1002/alz.091584

**Published:** 2025-01-03

**Authors:** Stefan Wendt, Ada J. Lin, Sarah Ebert, Declan Brennan, Wenji Cai, Brian Macvicar, Haakon B. Nygaard

**Affiliations:** ^1^ University of British Columbia, Vancouver, BC Canada

## Abstract

**Background:**

Murine models of Alzheimer’s Disease (AD) have resulted in numerous discoveries leading to a better understanding of AD pathogenesis but results poorly translated to novel treatment options. Over the past years, iPSC‐derived human neuronal cultures have been developed to better model AD *in vitro*. One key hallmark of AD is the presence of insoluble Aß plaques in the brain. To model this microenvironment a 3D tissue culture is needed to successfully study the complex neuron‐glia‐Aß interaction. Here we present human iPSC‐derived 3D neurospheres for modelling severe amyloidosis through chronic Aß application.

**Method:**

Neurospheres were formed from iPSC‐derived neuronal progenitor cells and expanded, differentiated and maintained for 60 days. Microglia were added to readily populate the tissue for the last 10 days of culture. Oligomeric Aß42 (∼35 nM) was added to the media chronically for 3 or 5 weeks. Neuronal activity was measured using GCAmP6f live imaging. Aß induced oxidative stress was quantified by using roGFP. Single nuclei RNA sequencing (NucSeq) was performed to obtain transcriptional data.

**Result:**

Neurospheres, consisting of neurons and astrocytes, display robust synchronized neuronal activity. Aß treatment induces the formation of plaque‐like aggregates in the tissue within days. We found dysregulated neuronal activity and oxidative stress after 2‐3 weeks while severe neuronal loss can be observed after 4‐5 weeks. Infiltrating microglia display high phagocytic activity towards Aß and successfully reduced extracellular Aß deposits, loss of neuronal activity and oxidative stress when added early during the Aß treatment. Microglia transferred late during Ab exposure ameliorated neuronal death but fail to recover neuronal function or reduce oxidative stress at this time point. NucSeq allows identifying the transcriptional profile of each cell type and will be used to study cell type specific changes after Aß exposure with or without microglia.

**Conclusion:**

We here present a human 3D cell culture model allowing the formation of neurospheres and a chronic Aß stimulation protocol to mimic amyloidosis as a novel human AD *in vitro* model. Transplanting microglia at different time points during culture allows to study the temporal dynamics of neuroprotective properties of microglia presenting a unique novel approach to reveal new potential therapeutic pathways.

